# The Cost of Managing Type 2 Diabetes Mellitus in Greece: A Retrospective Analysis of 10-Year Patient Level Data “The HERCULES Study”

**DOI:** 10.1155/2015/520759

**Published:** 2015-05-20

**Authors:** Ilias Migdalis, Grigorios Rombopoulos, Magdalini Hatzikou, Christos Manes, Nikolaos Kypraios, Nikolaos Tentolouris

**Affiliations:** ^1^NIMTS Hospital, 12 Monis Petraki Street, 11521 Athens, Greece; ^2^Novartis Hellas, 12th Km National Road 1, Metamorfosis, 14451 Athens, Greece; ^3^General Hospital of Thessaloniki “Papageorgiou”, West Ring Road, 56429 Thessaloniki, Greece; ^4^Polyclinic General Hospital, 3 Peireos Street, 10552 Athens, Greece; ^5^Laiko General Hospital, 17 Agiou Thoma Street, 115 27 Athens, Greece

## Abstract

*Objective.* This study aimed to estimate the mean annual cost of treating type 2 diabetes mellitus patients (T2DM) including complications and comorbidities in Greece.* Design.* A noninterventional retrospective study was based on patient level data analysis (bottom-up approach) from medical records, with at least 10-year-follow-up data.* Results.* The total annual cost per patient for managing diabetes in Greece was estimated at € 7,111 and was, statistically significantly, higher for patients with inadequate glycemic control (Hba1c > 7%) versus patients with adequate control (Hba1c = 7%) (€ 7,783 versus € 6,366, resp.; *P* = 0.017). This was mainly attributed to difference in CV hospitalizations between groups 14/111 versus 4/100, respectively, OR = 3.46 (95% CI: 1.10–10.9) for inadequately controlled patients. The largest component of cost was management of comorbidities, accounting for 48% of costs, and pharmaceutical treatment at 35.9% while only 14.9% was attributed to diabetes treatment per se. Obese men and patients with poor education are the groups with higher treatment costs.* Conclusions.* This is the first study to capture all cost components and the real burden of diabetes in Greece. Comorbidities were found to account for almost half of total cost, significantly higher in nonoptimally controlled diabetes patients.

## 1. Introduction

Diabetes mellitus (DM) is a chronic condition primarily defined by the level of hyperglycemia giving rise to risk of microvascular and macrovascular damage [1, 2].

Type 2 diabetes mellitus (T2DM) comprises 90% of people with diabetes around the world and is largely the result of excess body weight and physical inactivity [3]. A recent Greek study in a large representative rural, urban, and suburban population showed that T2DM was associated with advancing age, obesity, exposure to smoke, and low socioeconomic status [4].

T2DM has become an epidemic [5] and affects about 6% of the adult population in the western world [6]. In Greece, the projected prevalence of T2DM in 2002 was 7.6% in men and 5.9% in women [7]. Two other studies estimated the prevalence of diabetes among adult urban and rural populations in Greece: for the urban population it was estimated at 8.2% (men, 8.5%; women, 7.8%) in 2002 and 9.5% (men, 9.7%; women, 9.3%) in 2006 [8]; for the rural population, the prevalence of diabetes was estimated at 7.8% in 2002 [9].

There is an increasing trend in the prevalence of diabetes; the study by Wild and colleagues showed that the “diabetes epidemic” will continue even if levels of obesity remain constant [10]. Therefore, this trend becomes even more worrying since the prevalence of obesity, the primary risk factor of T2DM, also exhibits an increasing trend [11, 12].

Despite many advances in its treatment over the past few decades, T2DM remains a serious public health problem and is a growing burden on global economies [13]. It is associated with reduced life expectancy; in 2004, an estimated 3.4 million people died from consequences of high fasting blood sugar [14]. The World Health Organization's (WHO) projections show that diabetes will be the 7th leading cause of death in 2030 [15].

In addition, T2DM is associated with significant morbidity and low quality of life (QoL) due to specific diabetes-related microvascular complications, increased risk of macrovascular complications (ischemic heart disease, stroke, and peripheral vascular disease), blindness, renal failure, and amputations [1, 16, 17]. According to a recently published Greek study, patients with poorer glycemic control score significantly lower QoL levels compared to their well-controlled counterparts [18].

T2DM is also a very costly disease. The American Diabetes Association (ADA) estimated the total cost of diabetes in the US at $174 billion in 2007, including $116 billion in excess medical expenditures and $58 billion in reduced national productivity [19]. The total direct medical cost of T2DM in eight European countries was estimated at € 29 billion per year (at an average annual cost per patient of € 2,834) [20]. The INSTIGATE study showed that the mean total direct costs per patient in five European countries increased in the 6-month follow-up period, compared with the 6-month period prior to insulin initiation, and ranged from € 577 in Greece to € 1402 in France. In all countries, the breakdown of total direct costs by expenditure category varied considerably across countries, reflecting differences in resource use patterns, prices of medical resources, and different health care systems [21]. In Greece, the annual cost of treating diabetes had been estimated by Athanasakis and colleagues [22]. In addition, a more recent study estimated the mean costs associated with the management of T2DM, after initiating insulin therapy [23]. The aforementioned studies did not include hospitalization, comorbidities, and complications cost.

The primary objective of the study was to estimate the mean annual cost of T2DM treatment in Greece, based on medical records of patients with at least a 10-year history of T2DM. In addition, this study explored the association of total cost of diabetes with HbA1c levels, after controlling for a set of demographic and socioeconomic parameters, in order to identify the determinants and key cost drivers of diabetes.

## 2. Patients and Methodology

### 2.1. Description of Study Design

A noninterventional retrospective study was conducted between June 30, 2011, and June 1, 2012, in four diabetes centers operating in public hospitals. The four participating diabetes centers were among the 25 official diabetes centers in Greece and had sufficient databases with a patient follow-up of at least 10 years in order to be able to collect the necessary data retrospectively.

Patients were recruited during their routine visit for the management of their T2DM. Adult T2DM patients receiving any type of antidiabetic treatment at least for 10 years before recruitment who have performed at least one visit per year to the diabetes center throughout the last decade with a complete patient file and adequate medical data according to the evaluation schedule were considered as eligible to participate. Eligibility was assessed by the study investigators, who were medical staff of the aforementioned diabetes centers. Eligible patients willing to participate had to sign an informed consent in order to be enrolled. The study protocol was approved by the Institutional Review Board prior to study initiation (April 2011).

An electronic Case Report Form (eCRF) was developed in order to collect the data necessary for the analysis. The eCRF was completed for each of the patients only once and recorded data, based on patients 10-year medical history and on demographic characteristics, personal information, medical HbA1c measurements, complications of T2DM (all micro- and macrovascular complications), comorbidities, as well as resource use data (visits to physicians/outpatient visits, frequency and duration of hospitalization, pharmaceutical treatments, and laboratory tests), and work-loss days due to diabetes (absenteeism). Medical history data and laboratory test results were retrieved from medical records. If demographic or nonlaboratory data (absenteeism and number/duration of hospitalizations) were missing, they were collected through direct interviews with the patients conducted by the investigators during the same visit.

According to the study protocol, patients recruited in the study into two subgroups in a predefined 1 : 1 ratio, based on their mean HbA1c level over the last 10 years (at least one HbA1c measurement per year was a prerequisite). If the average HbA1c was equal or less than 7, the patient was categorized as adequately controlled, whereas if the average HbA1c was over 7, the patient was categorized as inadequately controlled. In order to achieve the 1 : 1 ratio, HBA1c measurement was uploaded in the eCRF system upon patient recruitment and participating investigators were informed on the number of patients recruited in each subgroup.

In order to estimate total costs for the management of diabetes and its comorbidities, unit costs were assigned to resource use data collected from patient files and related interviews. Pharmaceutical costs were retrieved from publicly available sources, hospitalization costs from Diagnosis Related Groups (DRGs) tariffs, cost for specialist visits, and examination costs from National Health Care System of Greece (NHS) price list [24–26]. Only direct costs were included in the study. All costs were estimated from the NHS perspective in 2013 prices (€).

### 2.2. Statistical Analysis

Statistical analysis was conducted with SPSS version 19.0. Descriptive analysis was used to describe the continuous and categorical data of patients. Bivariate and multivariate analyses were conducted to identify the sociodemographic and clinical parameters that mostly influence the cost, at a statistical significance level of 0.05. Bivariate analysis was performed using nonparametric tests (Mann-Whitney and Kruskal-Wallis). Due to the nonnormal distribution of the cost data, all costs were logarithmically transformed, which allowed for the use of parametric methods and resulted in regression models with better goodness of fit. The variables investigated in the bivariate and multivariate analyses were age, gender, disease control level (HbA1c), body mass index (BMI) at recruitment, comorbidities, and complications.

## 3. Results

### 3.1. Patient Characteristics

A total of 211 patients were enrolled in the study, of which 100 (47.4%) were categorized as adequately controlled and 111 (52.6%) as inadequately controlled. Patient characteristics were similar in the two subgroups with the exception of BMI and are presented in Table 1. Inadequately controlled patients were more likely to be obese compared to adequately controlled counterparts (*P* < 0.05).

Regarding comorbidities, patients with high HbA1c levels were more likely to suffer from hypertension but the difference was not statistically significant (88.3% versus 78.0%, *P* = 0.069). Moreover, the difference in the prevalence of all other comorbidities between the two groups was not statistically significant (Table 2).

The most common diabetes complications of study population were diabetic retinopathy (37%) and cardiovascular events (coronary artery disease including myocardial infarction and heart failure) (31%). Other complications included peripheral vascular disease (18%), diabetic neuropathy (17%), renal impairment (10%), and stroke (8.5%).

Regarding antidiabetic treatment, patients with high HbA1c levels were on average prescribed more medication than those in the adequately controlled group (*P* < 0.01). The duration of antidiabetic treatment was comparable between the two patient subgroups (*P* = 0.375) (Table 3).

There was a statistically significant difference (*P* < 0.001) in favour of patients on long and short-acting insulin of the adequately controlled group (Table 3). Differences in the use of all other antidiabetic agents (alpha-glucosidase inhibitors, biguanides, DPP4 inhibitors, glitazones, GLP-1 analogues, meglitinides, and sulphonylureas) were not statistically significant.

Only 46 (21.8%) of the 211 patients reported that they had been hospitalized during the period under consideration. Patients were on average admitted to hospitals 1.4 times per year for the treatment of diabetes and its complications, with a mean duration of hospitalization of 6.3 days. CV hospitalizations were 3.46 times greater (OR) for inadequately controlled patients (95% CI: 1.10–10.9) versus controlled patients with 14/111 (12.6%) and 4/100 (4%), respectively. CV hospitalizations (myocardial infraction, heart failure, by-pass, pulmonary edema, angioplasty, and stroke) and difference in stroke incidence between groups justify the total cost difference of controlled and uncontrolled patients. The total number and duration of hospitalizations were comparable between the two subgroups, with small differences not being statistically significant (Table 4).

Number of visits to a specialist in the diabetes center was on average 32.2 over the last 10 years, with no statistically significant differences between the two subgroups (*P* = 0.259). Although CV hospitalizations and stroke incidence were different between subgroups still the total number and duration of complications due to diabetes were not statistically significantly different between the two groups.

### 3.2. Costs

**Figure 1:**
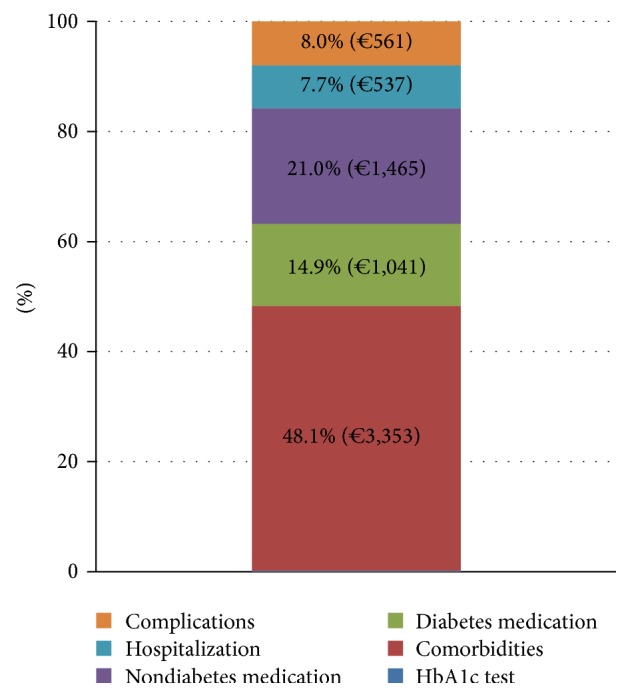
Breakdown of the total cost of diabetes management. Note: cost of HbA1c test is 0.2% (€ 13) of total cost; disability pensions are excluded.

The total annual cost per patient for managing diabetes was estimated at € 7,111 (SD = 4,323) excluding disability pensions. The largest component of this cost was management of comorbidities, accounting for 48% (€ 3,353) of total costs, while pharmaceutical treatment (including diabetes-related and nondiabetes medication) accounted for 35.9% (€ 2,506). Antidiabetic agents accounted only for 14.9% (€ 1,041) of total cost (Figure 1).

**Figure 2:**
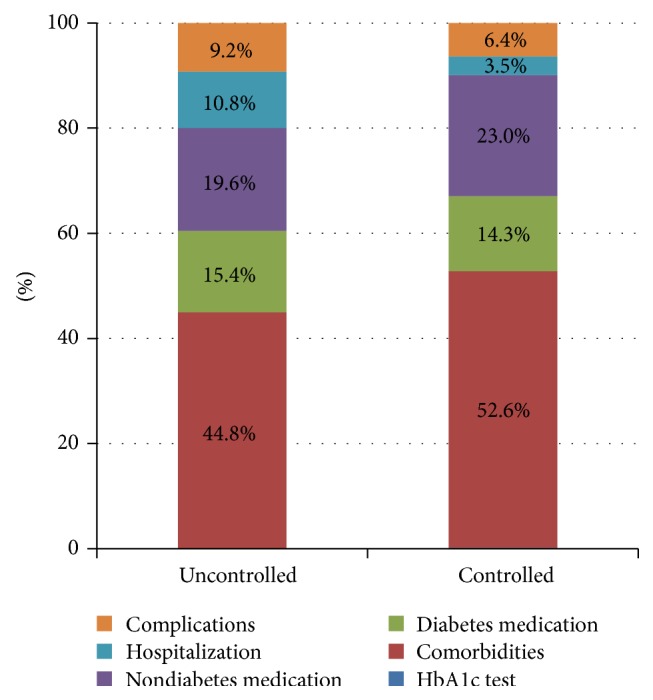
Breakdown of total cost of adequately versus inadequately controlled patients. Note: cost of HbA1c test is 0.2% of total cost in both subgroups; disability pensions are excluded.

For adequately controlled patients, the total cost per year was estimated at € 6,366, while the cost of inadequately controlled patients was estimated at € 7,783, with their difference being statistically significant (*P* = 0.017). Differences in costs of hospitalization and complications between the two subgroups were not statistically significant (*P* = 0.091).  Figure 2 presents the breakdown of adequately versus inadequately controlled patients' costs.

Bivariate analyses showed that inadequately controlled patients cost is on average € 1,417 more per year compared to the optimally controlled group (*P* = 0.017). Male patients showed higher costs than females (Table 5); the same applies to overweight or obese patients compared to normal weight. In addition, there was no statistically significant difference found between patient subgroups relating to employment, marital status, and monthly income level.

Existence of comorbidities vastly increases annual costs (*P* < 0.01). In particular, diabetes patients with coronary artery disease (CAD) were found to have the highest yearly cost (€ 11,662), followed closely by those with stroke (€ 11,366).

The relationship between the total cost of managing diabetes, the patient characteristics (demographics, personal information, and income level), and T2DM control level was explored through a multivariate analysis, with cost as a dependent variable and all other parameters as explanatory variables. The results of this analysis showed that healthcare for men with diabetes costs € 1,277 more per year, in comparison to healthcare for women with diabetes, and also that treating obese diabetics costs € 1,460 more per year in comparison to treating patients in other BMI groups. In addition, low education level (up to primary school) patients with diabetes cost € 2,341 more on average in comparison to better educated patients, while adding a year in the diabetes history of a patient increases his/her annual cost by almost € 90.

## 4. Discussion

The current study aimed to estimate the total cost of T2DM in Greece, based on a dataset of patients being followed up for at least 10 years. The main finding is that the average annual cost of diabetes treatment was € 7,111. The estimates of the total cost of diabetes are much higher than those reported by previous Greek studies. In particular, Athanasakis and colleagues estimated the annual per patient cost at € 1,297 per patient ranging from € 982 to € 1,566 for adequately controlled and inadequately controlled patients, respectively [22]. However, the latter study included only treatment cost and not costs of diabetic complications and comorbidities which, combined, are the largest component of the total cost (56%), as shown in the present study. For similar reasons, the current results deviate from the cost estimates of the study of Aloumanis et al., presenting a range between € 496 before initiation of insulin treatment and € 573 after initiating insulin therapy [23].

The current findings are in accordance with estimates of other international studies, which also show high per patient costs for the management of diabetes. In particular, the American Diabetes Association study estimated that people with diagnosed diabetes incur average medical expenditures of about $13,700 per year, of which about $7,900 is attributed to diabetes [27]. In addition, 23% of the medical costs are used to directly treat diabetes, while 50% of the costs are used to treat the portion of chronic complications that are attributed to diabetes [19]. This is comparable to our finding that treating comorbidities accounts for 48% of total costs.

The present study is the first one in Greece to have captured most of the cost components and therefore it may more accurately reflect the real burden of diabetes in the Greek health care setting. Estimating indirect costs were beyond the scope of this study; however, if indirect cost was included, estimates of total costs of T2DM would have been even higher.

The study also showed that the cost of treating inadequately controlled patients was statistically significantly higher (€ 7,783) than the cost associated with treating adequately controlled patients (€ 6,366), a finding which is also consistent with the literature [22]. From a recent published Greek study performed on 6,631 T2DM patients, the majority of the sample (59%) was inadequately controlled, leading to an additional burden of the national health care budget [18]. Another interesting finding of this study is that, concerning the cost of treatment, with the exception of the insulin, no statistically significant difference was observed between the two groups. This could be attributed to the legacy effect due to limited treatment options during the first decade of the diabetes history of study sample (mean T2DM duration about 20 years in the study population).

The current study had several limitations, one of which is the 10-year retrieval of data which might be underreported in patients file. Additionally, recall bias of such a long period might also be considered as a limitation. Based on the abovementioned, the lack of statistical significance in hospitalization and absenteeism between the two subgroups (adequately and inadequately controlled) could be partly attributed to recall bias arising from the fact that data on hospitalization were retrieved through interviews. The same applies to complication costs as that could be attributed to the retrospective design of the study. However, the abovementioned limitations could be considered as symmetric across the two subgroups and therefore of minimal effect.

## 5. Conclusions

This study is the first aiming to capture all cost components and better reflect the real burden of diabetes in the Greek setting. The cost of managing diabetes in Greece is high and is statistically significantly higher for inadequately controlled versus adequately controlled patients, attributed mainly to difference in CV hospitalizations and numbers of stroke. Obese men with a long diabetes history and patients with lower educational levels are the subgroups found to have higher treatment cost. The management of comorbidities constitutes a major cost component, accounting for 48% of total costs, while T2DM-related pharmaceutical treatment accounted only for 14.9%. Since comorbidities account for almost half of diabetes expenditure and inadequately controlled patients have significantly higher costs than adequately controlled, any efforts to contain diabetes burden should be aiming at preventing complications and comorbidities in order to reduce costs of managing diabetes.

## Figures and Tables

**Table 1:** Demographic characteristics.

	Total (*N* = 211)	HbA1c ≤ 7 (*N* = 100)	HbA1c > 7 (*N* = 111)	*P* value
Age (years)				
Mean (SD)	72.9 (8.1)	73.6 (7.5)	72.3 (8.6)	N/S
Gender				
Males	106 (50.2%)	51 (51.0%)	55 (49.5%)	N/S
Females	105 (49.8%)	49 (49.0%)	56 (50.5%)	
BMI (at time of recruitment)				
Normal/thin (<25)	37 (17.5%)	26 (26.0%)	11 (9.9%)	0.022
Overweight (25–30)	93 (44.1%)	43 (43.0%)	50 (45.0%)	N/S
Obese (>30)	81 (38.4%)	31 (31.0%)	50 (45.0%)	0.039
Years since first diagnosis				
Mean (SD)	21.2 (7.5)	20.0 (7.6)	22.3 (7.2)	N/S
HbA1c				
Mean (SD)	7.3 (1.0)	6.6 (0.4)	8.0 (0.8)	

BMI: body mass index; SD: standard deviation.

**Table 2:** Comorbidities.

	Total (*N* = 211)	HbA1c ≤ 7 (*N* = 100)	HbA1c > 7 (*N* = 111)	*P* value
Dyslipidemia	170/211 (80.6%)	80/100 (80.0%)	90/111 (81.1%)	0.739
Hypertension	176/211 (83.4%)	78/100 (78.0%)	98/111 (88.3%)	0.069
Coronary artery disease	51/211 (24.2%)	24/100 (24.0%)	27/111 (24.3%)	0.959
Stroke	15/211 (7.1%)	5/100 (5.0%)	10/111 (9.0%)	0.257
Other (not related to T2DM)	149/209 (71.3%)	71/99 (71.7%)	78/110 (70.9%)	0.897

**Table 3:** Pharmaceutical treatment: antidiabetic and comorbidities therapies.

	Total (*N* = 211)	HbA1c ≤ 7 (*N* = 100)	HbA1c > 7 (*N* = 111)	*P* value
Number of antidiabetic agents				
Mean (SD)	3.4 (1.5)	3.1 (1.4)	3.7 (1.5)	<0.01
Duration of antidiabetic therapy (years)				
Mean (SD)	7.6 (3.8)	7.1 (3.4)	8.1 (4.1)	0.375
Number of comorbidities treatments				
Mean (SD)	5.5 (3.0)	5.4 (3.2)	5.6 (2.8)	0.630
Antidiabetic agents (*n*)				
Alpha-glucosidase inhibitors	22/211 (10.4%)	15/111 (13.5%)	7/100 (7.0%)	0.187
Biguanides	180/211 (85.3%)	93/111 (83.8%)	87/100 (87.0%)	0.643
DPP4 inhibitors	59/211 (28.0%)	26/111 (23.4%)	33/100 (33.0%)	0.163
Glitazones	39/211 (18.5%)	23/111 (20.7%)	16/100 (16.0%)	0.481
GLP-1 analogues	9/211 (4.3%)	4/111 (3.6%)	5/100 (5.0%)	0.739
Long-acting insulin	129/211 (61.1%)	87/111 (78.4%)	42/100 (42.0%)	<0.001
Rapid-acting insulin	99/211 (46.9%)	68/111 (61.3%)	31/100 (31.0%)	<0.001
Meglitinides	45/211 (21.3%)	22/111 (19.8%)	23/100 (23.0%)	0.693
Sulphonylureas	137/211 (64.9%)	71/111 (64.0%)	66/100 (66.0%)	0.869

**Table 4:** Resource use associated with hospitalization, complications, and medical care.

	Total (*N* = 211)	HbA1c ≤ 7 (*N* = 100)	HbA1c > 7 (*N* = 111)	*P* value
Number of visits to a specialist^*^ per year				
Mean (SD)	3.2 (0.9)	3.1 (0.9)	3.3 (0.9)	0.259
Number of admissions to hospital per year				
Mean (SD)	1.4 (1.1)	1.2 (0.4)	1.5 (1.2)	0.528
Duration of hospitalization (days per hospitalization)				
Mean (SD)	6.3 (3.9)	6.6 (4.1)	6.2 (3.8)	0.831
Number of complications over the last 10 years				
Mean (SD)	2.1 (1.7)	2.2 (2.2)	2.0 (1.3)	0.732
Duration of complications (years)				
Mean (SD)	6.5 (4.1)	5.8 (3.6)	6.8 (4.4)	0.511

^*∗*^At the diabetes center.

**Table 5:** Average annual costs by patient subgroups.

	Mean annual cost per patient	SD	*P* value
HbA1c			
HbA1c > 7	7,783.3	4,549.0	0.017
HbA1c ≤ 7	6,366.6	3,948.7	
Gender			
Women	6,470.5	2,677.0	0.037
Men	7,747.2	5,428.5	
BMI			
Normal/thin (<25)	5,837.7	3,911.9	<0.01 (versus obese)
Overweight (25–30)	6,903.8	4,491.2	<0.01 (versus obese)
Obese (>30)	7,932.8	4,183.6	—
Education			
Primary school	8,104.2	4,649.9	—
Secondary school	5,640.2	2,575.1	<0.01 (versus primary)
High school	6,817.1	3,885.2	<0.01 (versus primary)
University	6,405.9	4,731.1	<0.01 (versus primary)
Comorbidities			
Dyslipidemia	7,889.6	4,290.1	<0.01 (versus CAD)
Hypertension	7,847.0	4,257.3	<0.01 (versus CAD)
Coronary artery	11,662.4	5,592.9	—
Stroke	11,366.4	8,389.2	<0.01 (versus CAD)
Other	7,699.8	4,349.3	<0.01 (versus CAD)

BMI: body mass index; CAD: coronary artery disease.
